# Logistic Regression and Linear Discriminant Analyses in Evaluating Factors Associated with Asthma Prevalence among 10- to 12-Years-Old Children: Divergence and Similarity of the Two Statistical Methods

**DOI:** 10.1155/2009/952042

**Published:** 2009-03-25

**Authors:** George Antonogeorgos, Demosthenes B. Panagiotakos, Kostas N. Priftis, Anastasia Tzonou

**Affiliations:** ^1^Department of Hygiene, Epidemiology and Medical Statistics, School of Medicine, University of Athens, 75 Mikras Asias Street, 11527 Athens, Greece; ^2^Department of Dietetics and Nutritional Science, Harokopio University of Athens, 70 El. Benizelou Street, 17671 Athens, Greece; ^3^Department of Allergy-Pneumonology, Penteli Children's Hospital, 15236 Palaia Penteli, Greece

## Abstract

Logistic regression and discriminant analyses are both applied in order to predict the probability of a specific categorical outcome based upon several explanatory variables (predictors). The aim of this work is to evaluate the convergence of these two methods when they are applied in data from the health sciences. For this purpose, we modeled the association of several factors with the prevalence of asthma symptoms with both the two methods and compared the result. In conclusion, logistic and discriminant analyses resulted in similar models.

## 1. Introduction

Logistic regression and linear discriminant analyses are multivariate statistical methods which can be used for the evaluation of the associations between various covariates and a categorical outcome. Both methodologies have been extensively applied in research, especially in medical and sociological sciences. Logistic regression is a form of regression which is used when the dependent variable is dichotomous, discrete, or categorical, and the explanatory variables are of any kind. In medical sciences, the outcome is usually the presence or absence of a stated situation or a disease. Using the logit transformation, logistic regression predicts always the probability of group membership in relation to several variables independent of their distribution. The logistic regression analysis is based on calculating the odds of the outcome as the ratio of the probability of having the outcome divided by the probability of not having it. Discriminant analysis is a similar classification method that is used to determine which set of variables discriminate between two or more naturally occurring groups and to classify an observation into these known groups. In order to achieve that discriminant analysis is based on the estimation of the orthogonal discriminant functions, the linear combination of the standardized independent predictor variables gives the greatest means differences between the existing groups. Thus, it can be proposed that both discriminant analysis and logistic regression can be used to predict the probability of a specified outcome using all or a subset of available variables.

Although the theoretical properties have been studied extensively throughout the literature, the choice of the proper method in data analysis is still a question for the researcher. The aim of this work after summarizing the properties of the two discriminating methods is to explore the convergence of the two analytical methods when they are used to evaluate categorical health outcomes in the pediatric epidemiological research. In particular, we tested the associations between anthropometric and lifestyle patterns in relation to asthma prevalence among 10–12-year-old children, using both statistical methods. So, the reader will elucidate the differences and the similarities of the two methods in order to make the appropriate choice in their application.

## 2. Material and Methods

### 2.1. Linear Discriminant Analysis

Discriminant analysis focuses on the association between multiple independent variables and a categorical dependent variable by forming a composite of the independent variables. This type of multivariate analysis can determine the extent of any of the composite variables discriminates between two or more pre-existing groups of subjects and also can derive a classification model for predicting the group membership of new observations [1]. The simplest type of discriminant analysis is when the dependent variable has two groups. In this case, a linear discriminant function that passes through the means of the two groups (centroids) can be used to discriminate subjects between the two groups. When there are more groups, the number of groups minus one function is needed to classify an observation among them. For each of the groups, linear discriminant analysis assumes the explanatory variables to be normally distributed with equal covariance matrices. For each case, the estimated coefficient for an independent variable is multiplied by the case's score on that variable. These products are summed and added to the constant, and the result is a composite score, that is, the discriminant score for that case.

The linear discriminant function (LDF) is represented by

(1) $$\text{LDF}=b_{0}+b_{1}x_{i1}+b_{2}x_{i2}+\cdots +b_{k}x_{ik}=bX,$$

where *b*_*j*_ is the value of the *j*th coefficient, *j* = 1,…, *k*, and *x*_*i j*_ is the value of the *i*th case of the *j*th predictor. The LDF can also be written in standardized form which allows comparing variables measured on different scales. In the standardized LDF, each variable is adjusted by subtraction of its mean value and division by its standard deviation. Coefficients with large absolute values reflect greater discriminating ability to their corresponding variables. From the LDF, scores can estimate predicted probabilities and predicted group membership for every case on the dependent variable. This approach is based on the rationale that it is more likely that the independent and dependent variables are related as the between-groups sum of square is larger relative to within-group sum of squares. Also the ratio of between-group divided by total sum of squares (eta-squared statistic or explained variability) or of within-group divided by total sum of squares (Wilks' lambda statistic or unexplained variability) is used to assess the relationship. As we can see, the ratio of between-group divided by within-group sum of squares is an analogue to the ratio of variances, which is the *F* statistic, a test that controls the possibility that the observed relationship is due to chance.

The principle by which the discriminant coefficients (or weights) are selected is that they maximize the distance between the two group means (centroids) $\left|{\bar{y}}_{1}-{\bar{y}}_{2}\right|$. Fisher [2] was the first who suggested to transform the multivariate observation *x* to univariate observations *y* in such way that the *y*'s derived from groups 1 and 2 have the maximum distance between them. Thus, the linear combination *y* = *a*′*x* is the one that maximizes the ratio (squared distance between sample means)/(sample variance *y*). The vector of coefficients is given by the eigenvectors of the matrix B*S ^−1^, where $B={\left({\overset{¯}{x}}_{1}-{\overset{¯}{x}}_{2}\right)}^{\prime }$ is the between-group matrix and *S* is an estimate of **Σ**. A very important characteristic of these composite sums of squares is that they enclose the variability and the covariability of each variable. The discriminant coefficients can be calculated in unstandardized or standardized form but they are irrelatively of the form, less informative than those in regression. Assuming that there are 2 groups, ${\overset{¯}{x}}_{1},\;{\overset{¯}{x}}_{2}$ are the means of each group, and *S* is the pooled covariance matrix, the allocation rule based on Fisher's discriminant functions is the following:

(2) $$X_{i}\in \{\begin{array}{cc} \text{group }1, & \text{if }y={\left({\overset{¯}{x}}_{1}-{\overset{¯}{x}}_{2}\right)}^{\prime }S^{-1}X_{i}\\ & \;\;\geq \frac{1}{2}{\left({\overset{¯}{x}}_{1}-{\overset{¯}{x}}_{2}\right)}^{\prime }S^{-1}({\overset{¯}{x}}_{1}+{\overset{¯}{x}}_{2}),\\ \text{group }2, & \text{if }y={\left({\overset{¯}{x}}_{1}-{\overset{¯}{x}}_{2}\right)}^{\prime }S^{-1}X_{i}\\ & \;\;<\frac{1}{2}{\left({\overset{¯}{x}}_{1}-{\overset{¯}{x}}_{2}\right)}^{\prime }S^{-1}({\overset{¯}{x}}_{1}+{\overset{¯}{x}}_{2}). \end{array}$$

### 2.2. Logistic Regression Analysis

Logistic regression is a form of regression which is used when we want to predict probabilities of the presence or absence of a particular disease, characteristic, or an outcome in general based on a set of independent of explanatory variables of any kind (continuous, discrete, or categorical) [3]. Since the predicted probability must lie between 0 and 1, simple linear regression techniques are insufficient to achieve that, because they allow the dependent variable to pass these limits and to produce inconsistent results. Defined as *P*_1_, the probability of an object is belonging to group 1, and as *P*_0_, the probability of an object is belonging to group 0. The logistic regression model has the form of

(3) $$\begin{array}{cc} z_{i} & =\log\left(\frac{P_{i1}}{P_{i0}}\right)\\ & =b_{0}+b_{1}x_{i1}+b_{2}x_{i2}+\cdots +b_{k}x_{ik}, \end{array}$$

where *P*_*i*1_/*P*_*i*0_ is called the odds ratio, *b*_*j*_ is the value of the *j*th coefficient, *j* = 1,…, *k*, and *x*_*i j*_ is the value of the *i*th case of the *j*th predictor. The parameters (*b*_*o*_ to *b*_*k*_) of the logistic model are estimated with the use of maximum likelihood method. The probability of an event to occur can be calculated using the logistic regression model

(4) $$P(Y_{i}=1\setminus X_{i})=\frac{e^{b^{T}X_{i}}}{1+\left(e^{b^{T}X_{i}}\right)}=\frac{1}{1+e^{-b^{T}X_{i}}},$$

where *e* ^*b*^*T*^*X*_*i*_^ is the linear predictor of the logistic regression function, and *Y*_*i*_ is the event under study (dependent variable).

If we use a probability cutoff of .5, then we can classify an object to group 1 if the estimated *P*_1_ > .5 and to group 0 if *P*_1_ < .5. In order to estimate the parameters of the logistic regression model, the method of maximum likelihood maximizes the coefficients of the log-likelihood function, a statistic which summarizes the information of the predictor variables.

Both logistic and linear discriminant regression analyses have the same functional frame; a composite of the independent variables and a rule for classification. But there are many differences about the assumptions made in order to apply them in a dataset.

Regarding discriminant analysis, the assumptions have great similarity with the assumptions made for ordinary regression and are (i) independent variables must have a multivariate normal distribution, thus allowing only continuous or ratio variables to enter the analysis and excluding all the forms of categorical variables, (ii) the variance-covariance matrix of all the independent variables must be homogenic among the population groups divided by the dependent variable (assumption that is controlled with several statistics, such as Box's *M *test), and (iii) independence of the cases.

Accurate estimation of the discriminant function parameters demands sample size of minimum 20 cases for each predictor variable and at least 20 cases for each of the dependent variable groups, otherwise the estimation of the coefficients is unstable and might lead to misleading results. The dependent variable in a discriminant analysis should be categorical, dichotomous, or polytomous. The population groups of the dependent variable should be mutually exclusive and exhaustive. Discriminant independent variables are assumed to be continuous. When categorical variables are included in the analysis, the reliability of discrimination of the analysis decreases [4, 5]. Discriminant analysis is highly sensitive to outliers. Lack of homogeneity of variances may indicate the presence of outliers in one or more groups. Lack of homogeneity of variances will mean that significance tests are unreliable, especially if sample size is small and the split of the dependent variable is very uneven. Lack of homogeneity of variances and presence of outliers can be evaluated through scatterplots of variables.

Logistic regression also has many limitations. At first, logistic regression assumes that there is an *s*-shaped dependency between the probabilities of group memberships and a linear function of the predictor variables. It also makes the assumption of independency among the observations. Analysis of the residuals may reveal patterns that indicate the presence of multicolinearity or can identify outliers, which can distort the valid estimation of the logistic coefficients. Also in order for logistic regression to give trustworthy and reliable estimates, it requires a large number of cases. The more unequal groups are formed from the dependent variable, the more cases are needed. On the other hand, logistic regression does not demand multivariate normality or homoscedasticity for the predictor variable, but if these conditions are fulfilled, the power of the prediction is increased [6, 7]. As in OLS regression, outliers can affect results significantly. The researcher should analyze standardized residuals for outliers and consider removing them or modeling them separately. Also, unlike OLS regression, logistic regression uses maximum likelihood estimation (MLE) rather than ordinary least squares (OLS) to derive parameters. MLE relies on large-sample asymptotic normality which means that reliability of estimates declines when there are few cases for each observed combination of independent variables.

For the evaluation of the two methods, sensitivity, specificity, and accuracy will be also measured in the same dataset. Sensitivity of a binary classification test with respect to some class is a measure of how well this test identifies a condition and expresses the probability of a case being classified in that class, meaning the proportion of true positives of all positive cases in the population. Specificity, on the other hand, expresses the proportion of the true negative classified cases of a binary classification test of all the negative cases in the population. Finally, accuracy is a measure of the degree of conformity of a measured or calculated quantity to the actual value. It is calculated as the proportion of the true results of a binary classification test (true positive and true negative) among all possible results.

Thus, linear discriminant analysis and logistic regression can be used to assess the same research problems. Their functional form is the same but they differ in the method of the estimation of their coefficient. Discriminant analysis produces a score, similar to the production of logit of the logistic regression. Both methods with the appropriate mathematical calculations produce the predicted probability of the classification of a case into a group of the dependent variable, and with the use of the appropriate cutoff value, we can also produce the predicted category of each observation. When categorical variables are entered in the analysis and are discrete measured, only the ones with large number of categories, more than 5, approximate the mean and the variance of the variables considered continuous and can be assumed to be normally distributed. Thus, the assumption of normality is fulfilled, and discriminant analysis makes robust estimations. On the contrary, logistic regression always produces robust estimations as it makes no assumption about the distribution of the explanatory variables or the linear relationship of them with the dependent variable and the equality of the variance within this group. So, when the assumptions of the discriminant analysis are violated, we should always avoid the discriminant analysis and analyze our data with logistic regression, which gives robust results since it can handle both continuous and categorical variables [8].

### 2.3. Application

#### 2.3.1. Use of Epidemiologic Data to Evaluate the Prevalence of Asthma

In the following study, we compared the results of discriminant and logistic regression analyses in predicting the presence of any asthma symptoms among Greek children aged 10–12 years old living in urban environment. During 2005, 700 students (323 males and 377 females), aged 10–12 years (4th–6th grade), were selected from 18 schools located in several areas of Athens, randomly selected from a list of schools provided by the regional education offices. The participation rate of the study was 95%. In order to evaluate asthma symptoms in the study sample, the parents completed seven questions according to the ISAAC protocol [9]. Particularly, the evaluation of the presence and the duration of asthma symptoms was assessed by four questions: (i) if children ever had wheezing, (ii) if they ever had disturbed sleep due to wheezing, (iii) if they ever had asthma, and (iv) if they ever had dry cough at night, except in cases of cold or chest infection. Further details about the data used may be found elsewhere [10]. The independent variables that were associated at a significance level *a* = 0.05 with the independent variable “presence of any asthma symptoms” were entered in a principal components analysis (PCA). Eighteen variables were fulfilling the above criterion, so 18 principal components were extracted from the analysis. Applying Kaiser's criterion (eigenvalue >1), we retained 8 factors, all mutually independent.

In order to examine the relationship between childhood asthma and the patterns that are extracted from PCA, the retained 8 components (patterns) were the predictor variables that entered in both discriminant and logistic regression models. The assumptions for the two models were all fulfilled (the components due to their extraction methods follow the multivariate normal distribution and are mutually independent), and variance covariance matrices of the groups were equivalent—Box's *M* test of equivalence *P*-value >.05—for the discriminant analysis, independency of the predictors, absence of multicolinearity after residual checking, and large number of observations for logistic regression model. We used the standardized canonical discriminant function coefficients and the unstandardized function coefficients for discriminant analysis and *Z* statistic (squared Wald statistic) for logistic regression, to evaluate how much each one of the variables contributes to the discrimination between two groups. The contribution of the respective variables to the discrimination depends on how large the coefficients are. We also compared the sign and magnitude of coefficients. Box's *M* test was used to check the equality of the covariance matrices, and it was revealed that they were equal (*P* > .05), thus this assumption for discriminant analysis was met.

For each model, we plotted the corresponding response operating characteristics (ROC) curve. An ROC curve graphically displays sensitivity and 100% minus specificity (false positive rate) at several cutoff points. By plotting the ROC curves for two models on the same axes, one is able to determine which test is better for classification, namely, that test whose curve encloses the larger area beneath it. All analyses were performed using the SPSS version 13.0 software (SPSS, Inc., Chicago, Ill, USA).

## 3. Results

**Figure 1:**
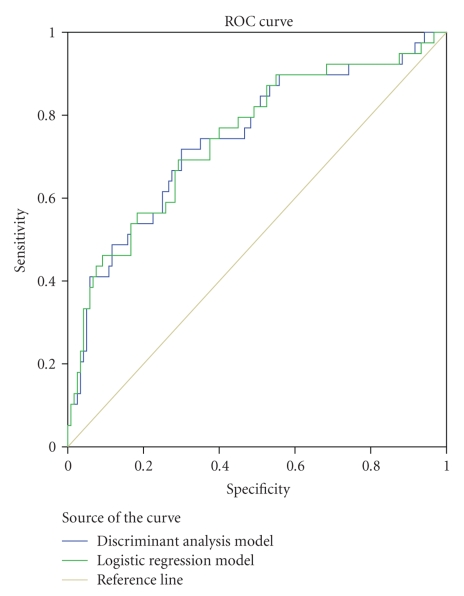
Receiver operating characteristics (ROC) curves for the discriminant analysis and logistic regression models.

Using PCA and applying Kaiser's criterion, 8 patterns of our original data were extracted, expressing the anthropometric indexes of the children, breakfast consumption, frequency of consuming athletic refreshments, parental anthropometric indexes, shortness of breath during recreational activities, birth weight and breastfeeding, eating cheese pies, and frequency of listening to music. These variables were used in both discriminant and logistic regression analyses, and both techniques revealed that anthropometric characteristics, athletic refreshment consumption frequency, and eating cheese pies were the most important contributors (Table 1). Moreover, we observe that the direction of the relationships was the same, and there were not extreme differences in the magnitude of the coefficients. The overall correct classification rate was 77.4% for discriminant analysis and 79.2% for logistic regression analysis. Table 2 presents sensitivity, specificity, and accuracy of both approaches at various cutoffs of the probability of having any asthma symptoms. Although some differences are observed between the methods, as we can see in Figure 1, the ROC curves of the aforementioned models clearly indicate that the logistic model is similar to the discriminant analysis model (i.e., no difference in the area under the curve (AUC), 74.6% versus 74.4%, *P* = .9).

## 4. Discussion

In general, both logistic regression and discriminant analyses converged in similar results. Both methods estimated the same statistical significant coefficients, with similar effect size and direction, although logistic regression estimated larger coefficients overall. The overall classification rate for both was good, and either can be helpful in predicting the possibility of a child having asthma symptoms in the general population. Logistic regression slightly exceeds discriminant function in the correct classification rate but the differences in the AUC were negligibly, thus indicating no discriminating difference between the models.

Discriminant analysis can use as a dependent variable a categorical variable with more than two groups, usually three of four. The number of the predicted discriminant functions equals with the number of the variable's categories minus 1. All of them have different sets of coefficients and produce a discriminate score for each case, but they have different classification ability. So, for a four level categorical dependent variable entering discriminant analysis, three discriminant functions are derived with their correspondent scores, and only one or two have the necessary power to achieve the optimum classification rates. The question arises in this case is about the number of functions which is needed to retain from the available set of functions.

In their paper, Brenn and Arnesen [11] compared the ability of discriminant analysis, logistic regression, and Cox model when applied in a dataset of 6595 men aged 20–49, who were followed for 9 years for total and coronary deaths, in order to select possible risk factors. People in the population sample were divided into two groups, one with mortality 5 per 1000 and one with 93 per 1000. Logistic regression and Cox model derived the same set of variables, and discriminant analysis set of variables had only minor differences. The researchers also noticed that a time-saving option, offered for both the logistic and Cox selection, showed no advantage compared with discriminant analysis, since by analyzing more than 3800 subjects, the logistic and Cox methods consumed, respectively, 80 and 10 times more computer time than discriminant analysis. Thus, the researchers reached to the conclusion that discriminant analysis is preferred for preliminary or stepwise analysis, otherwise Cox method should be used.

In the study of Pohar et al. [8], which used several simulated datasets and discrimination indexes, the convergence of the two methods is examined when the linear discriminant assumptions for normality of the distribution of explanatory variables are met, when they are violated, and when they are categorized for various parameters of the explanatory variables such as sample size, covariance matrix, Mahalanobis distance, and the direction of the distance between the group means. The authors concluded that linear discriminant analysis is a more appropriate method when the explanatory variables are normally distributed. For categorized predictor variables, linear discriminant analysis remains preferable, and logistic regression overcomes discriminant analysis only when the number of categories is small (2 or 3). When the assumptions of linear discriminant analysis are not met, the usage of it is not justified, while logistic regression gives good results regardless of the distribution of the predictors. In a study by Montgomery et al. [12], who compared the two methods in veterinary data using stepwise linear discriminant analysis and logistic regression in a first dataset and comparing the selected variables, the order of selection and the sign and the magnitude of the estimated coefficients of the discriminating models in a second dataset, resulted that although both methods converged logistic regression is preferable to discriminant analysis particularly when the assumptions of normality and equal variance are not met.

In order to compare the two methods, we applied them in a real dataset, and we did not use simulation methods, as the number of the observations in the dataset, although not very large, was sufficient to provide reliable results. Also, although linear discriminant function is a better method than logistic regression when the normality assumptions are met, the differences between them become negligible when the sample size is large enough (50 observations or more).

## 5. Conclusion

In conclusion, logistic regression and discriminant analyses were similar in the model analysis. In order to decide which method should be used, we must consider the assumptions for the application of each one.

## Figures and Tables

**Table 1:** Predictors, standardized, and unstandardized coefficients for the discriminant analysis model and logistic regression model.

Predictors	Logistic regression		Discriminant analysis	
	*b* coefficients	*Z* statistic	Unstandardized coefficients	Standardized coefficients
Anthropometric characteristics	0.529	2.676	0.325	0.319
Breakfast eating frequency	0.005	0.01	−0.011	−0.011
Athletic refreshments frequency consumption	−0.615	2.784	−0.459	−0.449
Parental BMI	0.268	1.397	0.103	0.103
Shortness of breath during activities	0.237	1.162	0.148	0.148
Birth weight and breastfeeding	−0.289	1.37	−0.182	−0.182
Cheese pies eating	0.355	1.695	0.226	0.225
Listening to music frequency	−0.294	1.393	−0.126	−0.126

**Table 2:** Sensitivity and specificity of logistic regression and discriminant analysis models, at various cutoff points for the probability of having any asthma symptoms.

Cutoff value*	Logistic regression			Discriminant analysis		
	Sensitivity (%)	Specificity (%)	Accuracy (%)	Sensitivity (%)	Specificity (%)	Accuracy (%)
.05	94.9	8.3	29.6	100	0.8	25.1
.10	92.3	23.3	40.2	100	1.7	25.8
.25	69	69.2	69.2	92.3	19.2	37.1
.50	28.2	95.8	79.2	71.8	70	70.4
.75	5.1	100	76.8	25.6	95	78
.90	0	100	75.5	5.1	100	76.8

*P (asthma symptoms): values less than or equal to the cutoff value indicate that the child is not having any asthma symptoms; those greater than the cutoff value indicate that a child is having one of asthma symptoms.
